# Homestay Hosting Dynamics and Refugee Well-Being: Scoping Review

**DOI:** 10.2196/58613

**Published:** 2024-11-25

**Authors:** Areej Al-Hamad, Yasin Mohammad Yasin, Kateryna Metersky, Sepali Guruge, Grace Jung, Khadija Mahsud

**Affiliations:** 1 Toronto Metropolitan University Toronto, ON Canada; 2 University of Doha for Science and Technology Doha Qatar

**Keywords:** homestay, host-guest relationship, hospitality, hosting, well-being, homestay accommodation, host-refugee relation, refugee, scoping review, review

## Abstract

**Background:**

Homestay accommodations aim to support a smoother transition for refugees; yet, the intricate nature of relationships between refugees and their hosting families can make this process complex, which, in turn, can affect their health and well-being. It is crucial to grasp the experiences of both refugees and their host families in order to foster effective settlement, integration, and well-being.

**Objective:**

The purpose of this scoping review is to explore the dynamics of homestay or hosting with a focus on understanding the experiences of both refugees and their hosting families to identify gaps in the literature and propose directions for future research.

**Methods:**

We used the Joanna Briggs Institute methodology and followed the PRISMA-ScR (Preferred Reporting Items for Systematic Reviews and Meta-Analyses extension for Scoping Reviews) checklist to guide this scoping review. Searches were conducted in MEDLINE via EBSCO, Scopus via OVID, CINAHL, SOCIndex, Web of Science Core Collection, ProQuest Dissertations and Theses, the SciELO Citation Index, and APA PsycInfo. Literature written in English and published from 2011 to 2024 that focused on homestay hosting contexts for refugees was included.

**Results:**

The results of this review illuminate the multifaceted and dynamic nature of homestay hosting for refugees. The findings include motivations and barriers for homestay hosting, factors influencing host-refugee relations, and psychological and social outcomes of homestay hosting.

**Conclusions:**

The results of this scoping review demonstrated the need for tailored support for refugees to improve homestay programs for the benefit of both refugees and host families and highlighted the need of more inclusive, supportive, and effective strategies for the hosting, resettlement, and integration of refugees.

**International Registered Report Identifier (IRRID):**

RR2-10.2196/56242

## Introduction

### Background

Refugee hosting encompasses a range of arrangements, such as permitting a displaced family to construct a shelter on the host family’s land, providing a portion of the home for a family’s use, cohabitating with a family in the same house or room, offering an outbuilding on the host’s premises for occupancy, and granting access to another property owned by the host for use by individuals [1,2]. Refugee homestay encompasses offering temporary shelter within the personal residences or properties of citizens and permanent residents of the host country and providing refugees with safety, shelter, and basic needs until they achieve independence or secure long-term accommodation [3-5]. This concept covers a spectrum of hosting options, from brief stays to longer-term arrangements, facilitated by grassroots volunteers, nonprofits, or governmental bodies [4] to address the social isolation and housing challenges refugees face upon arrival [4-6]. Hosting within the context of refugee resettlement frequently denotes an institutionalized social practice that appears to be embedded within a wider policy or humanitarian framework. Homestay extends the realm of private hospitality by functioning beyond migration networks, engaging primarily citizens of the host country who initially have no personal relationship with the refugees [4]. Refugee homestays, therefore, offer tailored support that eases refugees into their new environment, with host families being instrumental in facilitating refugees’ adaptation to local practices and integration into local communities within a nurturing, family-like atmosphere [4,7]. Within these contexts, refugees can take on roles that involve organization and collaboration with local residents, which can instill a strong sense of ownership and autonomy over their living conditions [8]. Thus, refugee homestays can also build social capital among refugees in their host communities [8,9].

Refugee homestays stand out as an economical approach that benefits both refugees and host community members [4,10]. This shared living setup offers a supportive environment for refugees, facilitating not only language learning but also the development of social networks that can provide essential support for refugees where they can leverage their cultural backgrounds [8]. Homestays provide a unique opportunity for deep cultural immersion, promoting intercultural dialogue [4]. This exchange enriches both refugees and hosts by sharing cultures, traditions, and values, thereby enhancing communal diversity and understanding [5]. Moreover, these housing arrangements open avenues for both refugees and local residents to learn and acquire new competencies through shared activities and interactions [4,10]. Understanding the dynamics of homestay hosting for refugees is vital for grasping its public health implications [11]. These living arrangements directly influence refugees’ access to health care, psychological support, and social integration, factors that are critical for maintaining physical and mental well-being [11]. By examining how homestays can shape health outcomes, we can better appreciate the necessity for supportive and informed health care policies tailored to the unique needs of refugees. Additionally, exploring health service accessibility and addressing the specific challenges faced by refugees in homestay environments could improve public health systems and outcomes.

Refugee homestay programs in Europe enhance refugee well-being through personal connections, diverse housing setups, and interactions with political landscapes, showcasing evolving solidarity amid immigration policies [4]. Refugee cohabitation and homestay programs play a critical role in improving the health and well-being of refugees by providing stable, supportive, and integrated living environments [12]. These arrangements foster a sense of belonging and community, which is crucial for mental health, reducing feelings of isolation and stress [12]. Access to shared resources and knowledge within these households can also improve refugee’s health by facilitating better nutrition and health care practices [13]. Moreover, the interpersonal relationships developed in homestays can offer emotional support and practical assistance, helping refugees navigate their new surroundings and the challenges of resettlement more effectively [12,13]. Hosting refugees can increase depression and mental health issues in hosts, possibly triggered by hearing refugees’ traumatic stories. This, combined with the stress of sharing living spaces and resources, can impact hosts’ mental health despite the altruistic benefits [14].

However, the informal nature of many homestay programs often results in power imbalances, with refugees seen as dependents on their hosts for a range of things [4]. Hosts’ well-intentioned support can also limit refugees’ interests and efforts at self-sufficiency [15], underscoring the delicate balance between assistance and independence within homestay interactions. In other words, homestay arrangements require considerable adjustments by both hosts and refugees, extending beyond mere accommodation to forming a domestic bond that encourages mutual respect and support [4,5]. However, intimate living situations can restrict refugees’ control over their personal space and privacy [15].

An initial search of the literature using review registries such as Joanna Briggs Institute (JBI) Evidence Synthesis and the Open Science Framework did not uncover any existing or ongoing scoping reviews on homestay or hosting dynamics and refugee well-being. The available literature reviews generally address the political issues that homestay accommodation for refugees raises in Europe [4] and the impacts of hosting forced migrants in lower-income countries [16]. In our study, we explicitly defined key terms such as “refugees” and “asylum seekers” to delineate the scope of our analysis. Refugees are “individuals forced to flee their country due to conflict or persecution,” while asylum seekers are “those seeking international protection but whose refugee status is yet to be determined” [17]. The concepts of “health” and “well-being” were explored in relation to physical, mental, and social factors, as outlined by World Health Organization guidelines [18]. Our review specifically targets the experiences of refugees in homestay settings, a focus chosen to uncover how these living arrangements impact health outcomes and integration processes. This approach can assist in exploring the unique challenges and supports that influence refugee well-being in nontraditional housing situations, addressing a significant gap in current public health research.

### Aim or Research Question

This scoping review aims to delve into the interactions between refugees and their host families. Scoping reviews allow for the collection, organization, and summarization of evidence [19] to identify trends, patterns, and differences and similarities on a phenomenon of interest. This scoping review aimed to answer the following research question: “What is known from the existing literature about the experiences of both refugees and their hosts within homestay or hosting dynamics that shape their health and well-being?”

## Methods

### Study Design

This scoping review was conducted following the JBI methodology for such reviews [19] and according to the PRISMA-ScR (Preferred Reporting Items for Systematic Reviews and Meta-Analyses extension for Scoping Reviews) checklist [19,20] (Multimedia Appendix 1). The protocol for this review has been registered with the Open Science Framework and has been published in *JMIR Research Protocols* [21].

### Inclusion and Exclusion Criteria

The inclusion and exclusion criteria are present in Textbox 1.

**Criteria for inclusion and exclusion of sources.**

**Inclusion criteria**

- Population: Records involving refugees, displaced individuals, or asylum seekers.
- Concept: Records on refugee homestay or hosting experience, homestay hosting practices, host-refugee relationship, and cohabitation.
- Context: Records related to refugee hosting across various host nations and geographical environments where homestay or hosting is practiced.
- Types of sources: Primary research papers, review papers, editorials, thesis and dissertations, reports, gray literature, conference presentations, and opinion pieces.
- Language: English.
- Publication date: Publications after 2011.

**Exclusion criteria**

- Population: Records involving a population other than refugees, displaced persons, or asylum seekers such as immigrants.
- Concept: Records on various hosting setups or focusing on hosting within a host nation or country that address different types of hosting experience.
- Context: Records on various hosting setups or focusing on hosting within a host nation or country.
- Types of sources: Incomplete and unrelated records that fall outside the review’s focus.
- Language: Language other than English.
- Publication date: Publication prior to 2011.

### Search Strategy

The search strategy was developed in collaboration with a research librarian based on seed papers provided by the lead researcher (AA-H) and an early search of various databases using fundamental terms. This strategy, incorporating all relevant keywords and index terms, was first applied to create a comprehensive search strategy for CINAHL, detailed in Multimedia Appendix 2. For other databases, the strategy was adjusted according to the specific Boolean operators, truncation, and wildcards. Additionally, our search strategy underwent peer review by another librarian using the PRESS (Peer Review of Electronic Search Strategies) Peer Review Strategy [22].

The review focused on studies published in English since 2011, and the reference lists of all selected sources were examined for further studies. The 2011 cutoff was selected because it marks a period of significant policy shifts and an increase in global displacement, leading to a surge in relevant literature on refugee health care needs and practices. This time frame ensures the inclusion of the most current and applicable findings since the Syrian refugee crisis. The databases searched encompassed Academic Search Complete, CINAHL, MEDLINE, SOCIndex via the EBSCO interface, APA PsycInfo via the OVID interface, the Web of Science Core Collection, ProQuest Dissertations and Theses, SciELO Citation Index via the Web of Science interface, and Scopus via their respective interfaces. Further search was performed using Google Scholar and a reference list of the selected papers. This approach considers the inclusion of gray literature, such as conference proceedings and opinion papers, to ensure the gathering of all relevant information related to the aim of this review and offer a comprehensive search and review.

### Study Selection and Extraction

Following the search, all identified citations were gathered and uploaded into EndNote (version 21; Clarivate), and the duplicates were removed. Titles and abstracts of the remaining sources were screened by 2 independent reviewers (AA-H and GJ) to assess their alignment with the review’s inclusion criteria. All relevant studies were retrieved, and their citation details were imported into the JBI System for the Unified Management of the Assessment and Review of Information for unified management, assessment, and review of information [23]. Two independent reviewers (AA-H and GJ) screened each title and abstract against the inclusion criteria. The full texts of selected citations were then assessed against the inclusion criteria by the 2 independent reviewers (AA-H and GJ). Reasons for exclusion of papers at the full-text stage, which did not meet the inclusion criteria, were recorded and reported. Any disagreements that arose between the reviewers at each stage of the selection process were resolved through discussion or with an additional reviewer (YMY). The outcomes of the search and the study inclusion process were reported and presented in a PRISMA (Preferred Reporting Items for Systematic Reviews and Meta-Analyses) flow diagram. Data were extracted from studies included in the review by the 2 reviewers (AA-H and GJ) using a data extraction table tailored to the content relevant to the review question (refer to Multimedia Appendix 3 [1-3,6,9,11,14,15,24-38]). The standardized data extraction tool from the JBI System for the Unified Management of the Assessment and Review of Information was used in this process, with minor adjustments made to align with the objectives of this review. The extracted data encompassed specific details about the participants, concept, context, study methods, and key findings pertinent to the review question. Any disagreements that arose between the 2 reviewers were resolved through discussion or with the third reviewer (KM). The team reached out to authors for further details or clarifications in cases of missing or ambiguous data.

### Data Analysis

The data analysis method used basic inductive content analysis, with 2 independent reviewers (AA-H and GJ) responsible for cataloging the characteristics of reports and the frequency of papers discussing refugee homestay arrangements, including the challenges, motivations, and outcomes identified. They then conducted a thorough review of the collected data (such as academic papers, reports, and other pertinent materials from a scoping review) to pinpoint significant codes pertinent to the paper’s population, concept, and context. These identified codes were then categorized into themes based on emerging trends, patterns, similarities, or differences. This coding process was both iterative and dynamic, allowing for the continual refinement and adjustment of codes, as additional data were reviewed [39]. To guarantee the reliability and precision of the coded information, a comparative method was used among team members to address any discrepancies either through team consensus or by consulting the third reviewer (KM) for resolution (see Multimedia Appendix 3 for the extraction table).

## Results

### Overview

**Figure 1:**
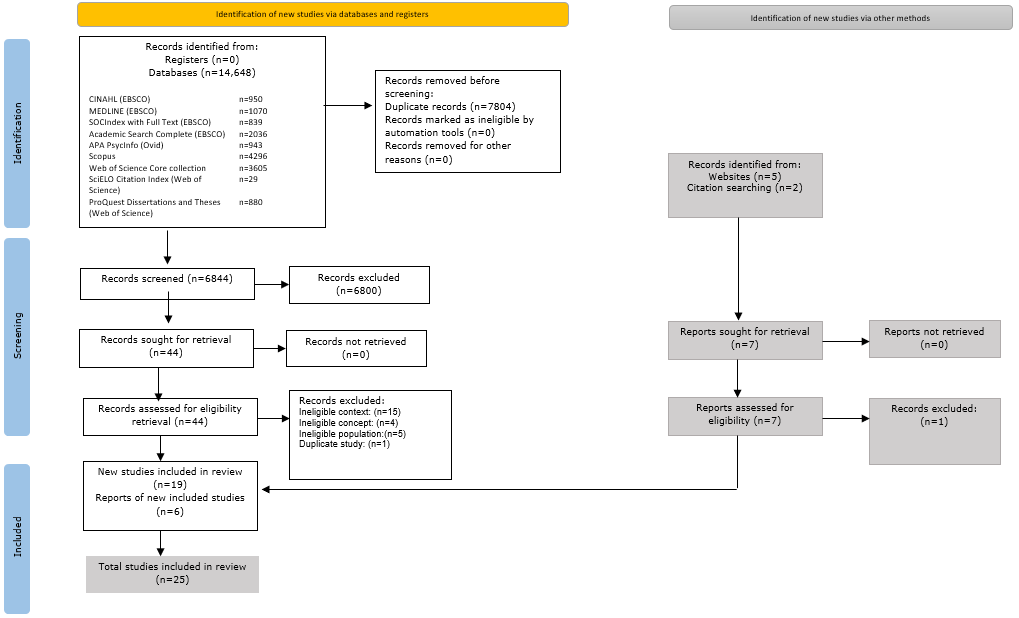
PRISMA (Preferred Reporting Items for Systematic Reviews and Meta-Analyses) diagram for selected studies.

The initial search resulted in 14,648 records for potential inclusion. Of these, 7804 duplicates were eliminated, along with 5 records that were identified as retracted (by Retraction Watch), leaving 6844 records for the first round of review, during which, the titles and abstracts were assessed for their relevance to the research aim, narrowing the field to 44 records for a detailed assessment of the full text. Of these, 15 were later excluded due to not fitting the context, 4 not aligning with the study’s concept, 5 not aligning with the study’s population, and 1 duplicate record. Following a specific search on Google Scholar and a manual check of references in the selected papers, an additional 6 records were added. In total, 25 records were deemed suitable for the final scoping review (Figure 1).

### Included Studies

**Figure 2:**
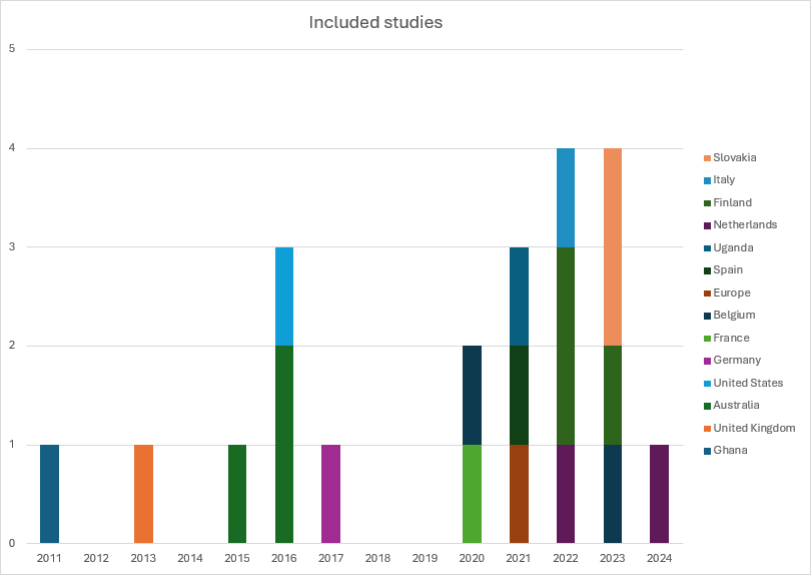
Geographical locations and years of publications for the included studies.

The papers included in this scoping review were published between January 2011 and January 2024. This scoping review included contributions from various geographical locations (see Figure 2 for the summary of the included studies) and spans a global array of research, encapsulating studies from across continents, including individual contributions from Ghana [24], the United Kingdom [25], Germany [26], the United States [14], Australia [27,28,40], Belgium [9,29], Finland [30-32], Slovakia [33,34], the Netherlands [11,35], Spain [6], Uganda [36], Italy [3], and a broader European perspective [37]. Alongside these studies, the review is enriched by 2 opinion papers [1,38] and 2 literature reviews [2,4]. This scoping review consists of blending empirical data from 4 quantitative studies [11,14,29,34], 15 qualitative studies [3,6,9,15,24-27,30-32,35-37,40], and 2 mixed methods studies [28,33]. The findings of this scoping review were categorized into (1) motivations and barriers for refugee homestay hosting, (2) factors influencing host-refugee relations in homestay hosting, and (3) psychological and social outcomes of refugee homestay hosting.

The sociopolitical contexts from which refugees emerge and the countries they migrate to play a pivotal role in shaping their homestay experiences. In Ghana, Liberian refugees encounter strained relationships with host communities due to resource allocation issues, land use, and perceived neglect by humanitarian organizations [24]. Similarly, in the Netherlands, displaced Ukrainian refugees experience variations in mental health outcomes based on the degree of their contact with host nationals, with factors like perceived discrimination paradoxically reinforcing their ethnic identity and, at times, improving their mental health in uncertain times like war and displacement [11]. In Slovakia, the hosting of Ukrainian refugees by local families demonstrates the role of hospitality in fostering social inclusion and building emotional ties, which in turn enhances societal cohesion [33]. However, the impact of such hospitality varies, with psychological resilience playing a crucial role in how well refugees can integrate and benefit from these arrangements. The Netherlands provides another illustrative example with the “TakeCareBnB” initiative, which has been effective in enhancing the integration of Syrian refugees by facilitating cultural and language learning and promoting deeper emotional and practical engagements with host families [35]. In the United Kingdom, the foster care system offers a range of relational dynamics from familial-like bonds to more formal guest-like interactions with young Afghan refugees, which significantly influence their sense of belonging and integration [25]. In Finland, hosts who live with asylum seekers from Syria, Iraq, and Afghanistan often experience deep emotional and sensory impacts. These interactions frequently go beyond simple hosting, drawing hosts into the refugees’ ongoing struggles and conflicts. As a result, the hosts’ own emotional landscapes are significantly influenced by the intense experiences and stories shared by the asylum seekers [30]. In France, the study on family hosting of refugees from Syria, Tibet, Bangladesh, and Zimbabwe shows improved language skills, cultural understanding, and overall well-being. However, it also points out issues like diminished independence and privacy concerns [15].

### Motivations and Barriers for Refugee Homestay Hosting

This theme aims to provide a comprehensive overview of the foundational elements that contribute to successful or strained homestay experiences, offering insights into how these factors can be managed or leveraged to promote better health outcomes within these unique settings. The theme examines the factors that influence the decision-making processes of both hosts and refugees as they enter homestay arrangements. The analysis delves into the complex motivations, such as cultural exchange alongside the perceived stability and safety for refugees. Concurrently, it explores barriers like cultural mismatches, language difficulties, and the psychological strains of adaptation that can impact these dynamics. Understanding these motivations and barriers is crucial, as they directly affect the health and well-being of refugees by shaping their living environments, social interactions, and access to community and health care services. Motivations for refugee hosting are often rooted in a deep sense of empathy, sympathy, compassion, opposition to the detention of refugees [27], social change, critique of government refugee policies and a desire to provide support to individuals in need [37], and volunteering [32]. Agblorti [24] illustrates that the Ghanaian hosts, despite facing significant sacrifices, express a sense of duty toward their refugee “brothers and sisters.” This sentiment of humanitarian concern is echoed in studies across different contexts, including the Netherlands [11] and Slovakia [33,34], where the social inclusion of refugees and the personal satisfaction from offering assistance are highlighted as key motivators. Furthermore, initiatives like TakeCareBnB in the Netherlands showcase how structured programs can facilitate these motivations, enhancing integration and fostering cultural and emotional support [35]. Overall, hosts’ motivation for hosting refugees often centers on compatibility, shared values, class, gender, cultural differences, and specific guest profiles such as “woman with a small child” [32]. People are often motivated to host due to a moral awakening sparked by media reports, prompting them to welcome refugees into their homes [30]. The role of social innovation and policy interventions emerges as a pivotal factor in mitigating barriers and enhancing motivations [4]. Media coverage significantly influences public views on refugees and homestay hosting, often casting refugees in a negative light through sensationalism and inaccuracies [40]. Such portrayals can foster hesitation about and resistance to welcoming refugees into their homes [40].

Barriers to hosting, on the other hand, introduce challenges that can deter or complicate the hosting experience. Caron [1] identifies the uncertainty regarding the duration of stay and the potential for overstaying as significant concerns for hosts, reflecting the anxiety associated with sharing living spaces over indefinite periods. The presence of children and economic constraints can create tensions among children or the strain of sharing resources leading to unsustainable relationships [2]. Studies from Australia [40] and Belgium [29], for example, highlight the challenges posed by cultural and language barriers as well as the psychological distress experienced by refugees, which can impede social integration and strain host-guest relationships (Table 1).

**Table 1:** Key motivations and barriers associated with refugee homestay hosting.

Aspect	Motivations	Barriers
Cultural exchange	Hosts motivated by the opportunity to engage in cultural exchange, providing stability and safety for refugees.	Cultural mismatches and language difficulties can create challenges, leading to strained interactions between hosts and refugees.
Empathy and compassion	Deep sense of empathy, compassion, and opposition to the detention of refugees. Motivations include social change, critique of government policies, and volunteering.	Psychological strains of adaptation, including stress and anxiety from living in a new environment, can impact refugees’ well-being and social integration.
Humanitarian concern	Sense of duty toward refugees, as seen in contexts like Ghana, where hosts make sacrifices out of humanitarian concern. Similar sentiments are echoed in the Netherlands and Slovakia, where social inclusion and personal satisfaction motivate hosts.	Uncertainty regarding the duration of stay and the potential for overstaying can cause anxiety and strain relationships. Economic constraints, especially in households with children, can exacerbate tensions and make hosting unsustainable.
Structured programs	Programs like TakeCareBnB in the Netherlands enhance motivation by facilitating integration and offering cultural and emotional support.	Media portrayal of refugees, often negative and sensationalized, can influence public opinion, leading to resistance or hesitation in hosting refugees.
Moral awakening	Moral awakening, often triggered by media reports, prompts individuals to welcome refugees into their homes.	Psychological distress experienced by refugees, such as trauma and stress, can impede their social integration and strain relationship with hosts.
Social innovation	Social innovation and policy interventions play a key role in mitigating barriers and enhancing motivations.	Challenges in sharing resources, especially in families with children, can create tensions and lead to unsustainable hosting arrangements.

### Factors Influencing Host-Refugee Relations in Homestay or Hosting

The theme “factors influencing host-refugee relations in homestay or hosting” focuses on identifying and analyzing the key elements that shape the dynamics between hosts and refugees in homestay settings. It explores how factors such as empathy, communication styles, expectations, and cultural sensitivity impact the relationship quality and, subsequently, the integration experiences of refugees. These relational dynamics are critical, as they directly influence refugees’ psychological comfort, sense of belonging, and overall mental health. By examining these factors, the theme aims to highlight practical ways in which host-refugee interactions can be improved to foster a supportive environment that enhances the health and well-being of refugees. The dynamics of host-refugee relationships are deeply influenced by factors such as cultural proximity, gender, and shared interests, and the sociopolitical context can significantly affect the overall experiences and outcomes of refugee homestay hosting. These significantly influence the outcomes of these arrangements. For example, Al-Saqaff [11] highlighted how the amount and type of host interaction affect displaced Ukrainian refugees, showing differences in integration shaped by gender and discrimination and enhancing the understanding of host-refugee dynamics. Additional factors that affect homestay accommodation include the unpredictability of how long guests will stay, the dimensions and placement of the hosting environment, the expenses related to accommodation, the risk of overcrowding due to family size, the impact of children’s presence and actions, and the financial standing of the host family [1,2].

Bassoli and Luccioni [4] offer critical insights into the factors shaping hosting experiences and the political implications of homestay accommodation such as the allocation of shared spaces and resources and participation in group activities. Alrawadieh et al [33] concluded that the direct contact between hosts and refugees allowed for a deeper understanding of the refugees’ backgrounds, facilitating a genuine connection and empathy. Moreover, factors like common interests, religious ties, cultural proximity, and gender play a significant role in influencing hosting behavior [33]. For example, shared religious beliefs or cultural practices can provide common ground, fostering a sense of connection and belonging [33]. Refugees faced difficulties adjusting to homestay arrangements and felt isolated without cultural connections, while hosts dealt with skepticism and disapproval from their social circles, complicating the hosting dynamic [27]. This aligns with findings from other studies [1,40] that have identified potential barriers including economic constraints, hosts’ uncertainty about the length of stay for accommodated refugees, cultural clashes, and the emotional toll of hosting, which can impact the durability of these relationships. Participation in household culture and the development of trusting and reciprocal relationships between hosts and refugees play a significant role in the dynamics of homestay hosting [25].

The close connection between refugees and their hosts is deepened by technology and the emotional toll of global conflicts [30]. Technology helps forge deep bonds and understanding by linking refugees’ past and current situations [30]. However, the risk of deportation introduces emotional and psychological challenges, impacting the safety and stability of the hosting environment [30]. Brinker [6] commented that intermediaries perceived by refugees as key facilitators in the hosting arrangement offer personalized support and easing coliving adjustments. However, intermediaries’ actions could sometimes exacerbate power imbalances and affect their neutrality due to close ties with hosts. Intermediaries also often stepped back once they deemed their assistance unnecessary, affecting the dynamics between hosts and refugees [6]. Radical cosmopolitanism transforms the host-refugee relationship from hierarchy to equality, focusing on mutual experiences and engagement rather than charity. It contests traditional divides, promotes equitable interactions, and fosters unity, thereby dismantling exclusionary barriers and enriching the host-refugee connection (Table 2) [26].

**Table 2:** Key factors influencing host-refugee relations in homestay settings.

Factor	Description	Impact on host-refugee relations
Empathy and communication	Effective communication styles and empathy are crucial for fostering supportive host-refugee relationships.	Enhances psychological comfort, sense of belonging, and overall mental health of refugees.
Cultural proximity	Similar cultural backgrounds, shared religious beliefs, or common interests between hosts and refugees.	Facilitates genuine connections, fosters a sense of belonging, and eases integration into the host environment.
Gender dynamics	Gender roles and expectations can vary significantly between hosts and refugees.	Affects integration experiences, with gender and discrimination playing a role in shaping the host-refugee dynamic.
Sociopolitical context	The broader sociopolitical environment, including the public’s attitude toward refugees and government policies.	Influences the ease or difficulty of hosting, with social disapproval or skepticism potentially complicating the hosting dynamic.
Household dynamics	Participation in household culture, including allocation of shared spaces and resources, as well as group activities.	Trusting and reciprocal relationships are crucial; failure to integrate into household culture can strain relationships and impact the success of the hosting arrangement.
Financial constraints	Economic limitations of hosts, including the cost of accommodating refugees and the potential for overcrowding.	Financial strain can lead to stress and tension, making it difficult for hosts to sustain long-term hosting relationships.
Duration of stay	Uncertainty about the length of the refugee’s stay.	Causes anxiety and can lead to strained relationships if the duration exceeds the host’s expectations.
Technology	Use of technology to maintain connections between refugees and their past or current situations.	Technology can deepen emotional bonds and understanding but also brings challenges related to the emotional toll of global conflicts and the risk of deportation.
Intermediaries	Role of intermediaries in facilitating the hosting arrangement.	Intermediaries can provide personalized support but may also introduce power imbalances and affect the neutrality of the hosting arrangement.
Radical cosmopolitanism	A philosophical approach that transforms host-refugee relationships from hierarchical to egalitarian, focusing on mutual experiences and engagement rather than charity.	Promotes equitable interactions, dismantles exclusionary barriers, and enriches the host-refugee connection, leading to more positive and sustainable relationships.

### Psychological and Social Outcomes of Refugee Homestay Hosting

The psychological and social outcomes of refugee homestay hosting are multifaceted, reflecting a complex interplay between personal interactions, societal perceptions, and individual experiences. The theme critically examines the mental health and social integration outcomes for refugees living in homestay arrangements. This theme explores how these living situations affect refugees’ psychological well-being, including aspects of stress, anxiety, and depression, as well as their social outcomes, such as community integration, social networks, and cultural adaptation. By assessing both the positive impacts, such as increased social support and cultural understanding, and the challenges, like isolation or cultural clashes, this theme provides a nuanced view of how homestay settings can influence refugees’ overall health and social well-being. The intent is to offer insights into how homestay environments can be structured and supported to promote better mental health and social integration, crucial for the long-term success and well-being of refugees.

The depth and quality of contact between refugees and hosts play a significant role in mental health outcomes, with increased contact generally correlating with better mental health, especially among female refugees [11]. The role of hospitableness in promoting refugee social inclusion cannot be overstated. Creating a welcoming environment that fosters emotional and cultural connections is essential for integrating refugees into society and improving their well-being [33,34]. Employment opportunities, in particular, have been highlighted as beneficial for refugees’ self-esteem and overall mental health, pointing to the critical need for access to work as part of the integration process [34]. The research by both Alrawadieh et al [33] and Altinay et al [34] underscores the importance of a welcoming atmosphere and emotional ties between hosts and refugees, highlighting the significant yet nuanced role of hospitableness in fostering societal cohesion and integration. van Dijk et al [35] in the Netherlands and Sirriyeh [25] in the United Kingdom explored innovative integration models and fostered care arrangements that emphasize the mutual process of integration, revealing the potential of such arrangements to enhance social connections, cultural understanding, and emotional support. Homestay hosting often hinges on overcoming prejudices and the ability of young refugees to integrate into household cultures [25].

Host-refugee relationships that are rooted in equality, everyday practices, and the breaking down of traditional barriers can potentially advocate for a society that values protection and support for refugees as a fundamental obligation, fostering a sense of togetherness and community integration [26]. Despite the positive aspects of homestay hosting, challenges, such as mental health risks for refugees and hosts, the potential for strained relationships, and issues of dependency and gratitude among refugees point to the need for comprehensive support mechanisms [27,40]. These mechanisms should aim to balance the benefits of homestay accommodation with the challenges it presents, ensuring that both hosts and refugees can navigate their relationships positively and productively [27,40].

The concept “asylumscapes” redefines the host-refugee relationship by highlighting the emotional labor and complex interactions involved, which significantly affect both psychological well-being and social cohesion [38]. Advocating for a better grasp of these relationships and hosting practices can nurture mutual support and improve social interactions, boosting both the resilience and inclusiveness of the communities involved [38]. Hosting asylum seekers offers an opportunity for compassionate engagement but also poses potential mental health risks for the hosts. The challenges of sharing limited spaces and resources, coupled with exposure to refugees’ traumatic stories, can contribute to increased stress and a heightened risk of depression and other mental health issues among hosts [14]. Finally, Bassoli and Campomori [3] emphasized the intercultural conversations that support the independence of guests to facilitate societal transformation while ensuring the integration of refugees, offering mutual advantages to both hosts and guests (Table 3).

**Table 3:** Key psychological and social outcomes of refugee homestay hosting.

Outcome type	Description	Impact on refugees and hosts
Mental health	The quality of contact between refugees and hosts significantly influences mental health outcomes. Increased contact generally leads to better mental health, especially for female refugees.	Positive interactions reduce stress, anxiety, and depression, while challenges such as dependency and shared space pressures can exacerbate mental health issues.
Social integration	Homestay hosting fosters social networks, cultural understanding, and community integration by creating emotional and cultural connections.	A welcoming environment promotes societal cohesion and integration, enhancing the well-being of refugees. Cultural clashes or isolation can hinder these benefits.
Employment opportunities	Access to employment boosts refugees’ self-esteem and overall mental health, making it a critical part of their integration process.	Employment provides a sense of purpose and belonging, improving mental health and facilitating social integration. Lack of employment opportunities can lead to frustration and social exclusion.
Hospitableness	Creating a welcoming and emotionally supportive environment is essential for integrating refugees and improving their well-being.	Hospitableness fosters positive relationships and societal cohesion, but the emotional labor involved can strain hosts, particularly when dealing with refugees’ traumatic experiences.
Cultural adaptation	The ability of young refugees to integrate into household cultures is crucial for the success of homestay arrangements.	Successful cultural adaptation leads to better social and emotional outcomes, while failure to adapt can result in isolation and strained relationships.
Equality and everyday practices	Relationships rooted in equality and mutual respect can break down traditional barriers, advocating for a society that values refugee protection and support.	Equality in host-refugee relationships fosters a sense of togetherness and community integration, promoting social cohesion and reducing feelings of dependency among refugees.
Dependency and gratitude	Feelings of dependency and gratitude among refugees can complicate relationships, leading to potential strain and mental health risks.	While gratitude can strengthen bonds, excessive dependency can create power imbalances and strain the relationship, highlighting the need for support mechanisms to manage these dynamics.
Asylumscapes	The emotional labor and complex interactions involved in homestay hosting redefine the host-refugee relationship, affecting both psychological well-being and social cohesion.	Understanding the emotional and social dynamics of asylumscapes can improve mutual support, resilience, and inclusiveness within host communities, benefiting both refugees and hosts.
Intercultural conversations	Intercultural conversations within homestay settings support the independence of guests and facilitate societal transformation, offering mutual advantages to both hosts and guests.	These conversations enhance cultural understanding and promote the integration of refugees, leading to positive social outcomes and reducing the potential for cultural clashes.
Mental health risks for hosts	Hosts may experience mental health risks due to the pressures of shared spaces and resources as well as the emotional toll of dealing with refugees’ traumatic experiences.	Hosts may develop symptoms of depression or other mental health issues, underlining the importance of providing support to manage the emotional challenges of hosting.

## Discussion

### Principal Findings

This scoping review included 25 papers that were published between 2011 and 2024 that met the inclusion criteria. As the world continues to adapt to the persistent refugee crises, comprehending the nuances of shared living arrangements is crucial for creating efficient and enduring solutions [41]. Collaborative efforts involving humanitarian organizations, nongovernmental agencies, and host countries are essential in providing displaced populations with protection, vital services, and opportunities for rebuilding their lives [42] in support and leaving many refugees in precarious housing situations [43]. The review concluded that refugee homestay or hosting presents a unique opportunity to foster societal cohesion, cultural exchange, and mutual support between hosts and refugees. This finding is consistent with other studies, where refugee homestay and shared living spaces not only serve as a practical solution to resource limitations but also create a distinctive chance for refugees to exchange cultural experiences, combine their methods of coping, and collaboratively deal with the complexities of resettling [44,45]. By providing sustained support to hosting families and communities, stakeholders can contribute to the creation of a connected and resilient global network [46]. Such international collaboration mitigates the risk of individuals feeling isolated or disconnected, fostering an environment where refugee resilience can flourish as a collective outcome [47].

The sociopolitical context of the countries involved in the reviewed literature plays a crucial role in shaping the experiences of both refugees and hosts in homestay arrangements. In countries like Germany [26], the United Kingdom [25], and the Netherlands [11], the political discourse surrounding immigration and refugee policies has been highly polarized, often influenced by media narratives that oscillate between humanitarian empathy and security concerns. For instance, media representations in these countries may emphasize either the vulnerabilities of refugees or the potential risks they pose, thereby influencing public perceptions and, consequently, the experiences of both hosts and refugees in homestay settings. In countries like Slovakia [33] and Finland [30], where sociopolitical contexts may be less diverse, the experiences of refugees in homestay arrangements might be shaped by a stronger emphasis on cultural assimilation, with hosts potentially feeling a greater responsibility to “integrate” their guests into the local way of life [43]. Conversely, in countries such as Uganda [36] and Ghana [24], where communities may have a history of displacement and resilience, the sociopolitical environment may foster a more community-oriented approach to hosting, although economic constraints often pose significant challenges. The sociopolitical climate in the United States [14] and Australia [28], where immigration is a contentious issue, can lead to highly varied experiences, with some hosts motivated by a strong sense of social justice and others influenced by more conservative, security-focused narratives. These media-driven narratives and public perceptions inevitably impact the dynamics of homestay arrangements, shaping the motivations, interactions, and outcomes for both refugees and hosts [27,31].

Nevertheless, the sustainability of positive host-refugee relationships demands ongoing support to navigate the complex dynamics and challenges that arise over time. The psychological and social impacts of hosting are multifaceted, reflecting the need for comprehensive support mechanisms to balance the benefits and challenges of homestay accommodation. This finding is coherent with the findings of Baggio [48] that highlight the profound loss of homes and disruption of social structures amplifying the psychological distress experienced by refugees and underscoring the need for comprehensive support mechanisms. In addition, the resulting state of refugees’ constant unfamiliarity and instability exacerbates the psychological distress stemming from the original conflicts that forced them to flee [15,32]. Through a deeper exploration of these challenges and stressors, scholars and policy makers can contribute to refining coliving as a viable pathway for displaced individuals to not only survive but thrive in their pursuit of a secure and settled life [49].

Recognizing that the responsibility for successful settlement extends beyond borders, international stakeholders, organizations, and agencies directly engaged in these matters must spearhead a concerted effort to ensure that homestay refugee hosting emerges not just as a plausible but as a widely adopted and effective solution on a global scale [42,46]. By acknowledging the complexities inherent in these shared living spaces, policy makers, humanitarian organizations, and host communities can work together to create environments that foster dignity, inclusivity, and hope for those rebuilding their lives in the wake of displacement [49]. Our study’s findings shed light on key aspects of homestay hosting for refugees, offering actionable insights for enhancing public health strategies and interventions. By illustrating the direct impacts of homestay environments on refugee health outcomes, these insights encourage the development of tailored health services that address the unique needs of refugees in such settings. However, the research also highlights significant gaps in the literature, particularly around safe and secure service practice guidelines for homestay hosting, the long-term psychological effects of homestay arrangements, and the sociocultural dynamics between hosts and refugees. Future research should focus on these areas to refine understanding and inform more effective policy formulation. This deeper exploration is crucial for crafting interventions that not only support refugee health in the immediate but also contribute to their long-term well-being and integration into new communities.

Our review explicitly details the cultural and adjustment challenges faced by refugees, underscoring how these factors significantly influence their health and well-being. By examining the nuanced interactions between refugees and their host environments, we highlight the critical role of cultural congruence and social support in the adjustment process. These elements are pivotal in shaping refugees’ mental health and overall well-being, as they navigate the complexities of integrating into new societies. Understanding these dynamics can inform the development of targeted interventions that address specific cultural and adjustment needs, ultimately improving health outcomes and facilitating smoother transitions for refugees in their host countries.

### Implications

The insights from this review are intended to guide the development of more inclusive, supportive, and effective approaches to refugee hosting, resettlement, and integration, with a strong emphasis on public health implications. The findings extend beyond academic discussion, offering practical guidance for policy makers, public health practitioners, and researchers. From a public health perspective, service providers and health care professionals working with refugees can leverage our findings to implement more inclusive and supportive strategies that address the unique health and well-being needs of refugees and their host families [50]. For example, social workers and community organizers can develop tailored programs that promote cultural exchange, language learning, and social integration, all of which are crucial for mental health and social cohesion. The review underscores the necessity of developing customized support mechanisms that mitigate the health challenges inherent in homestay arrangements. Public health practitioners should focus on creating environments that empower refugees, fostering a sense of belonging and community, which are essential for overall well-being [4]. Ultimately, our aspiration is that our findings will inform the establishment of more individualized and compassionate support practices within public health frameworks, ensuring that both refugees and their host families can thrive together in a healthy and supportive environment.

From a policy perspective, policy makers are encouraged to develop clear, actionable policy guidelines that support the creation of more effective and compassionate refugee homestay hosting models. These policies should aim to streamline the resettlement and integration process, ensuring that refugees and their host families have the necessary resources and support. Additionally, further investigation into the long-term impacts of homestay arrangements on refugees and their hosts highlights the need for policies that are not only responsive but also sustainable to ensure support for refugees and host communities alike [3,51]. By advocating for customized support mechanisms, explicit policy directives, and further investigation into the enduring impacts of homestay arrangements, our review laid the groundwork for the conduct of subsequent studies to expand upon our findings.

From a research perspective, the review serves as a foundation for future research in the field, encouraging scholars to explore the nuanced dynamics of refugee hosting. By identifying gaps in the current literature, the review invites further exploration of how homestay arrangements affect the well-being of refugees as well as hosts over time. We also recommend that future research investigates the effectiveness of different hosting models, with the goal of informing best practices for refugee support. This includes studying the impact of various support mechanisms on the integration process and identifying strategies that facilitate positive outcomes for both refugees and host families.

### Strengths and Limitations

To the best of our knowledge, this review is the first to explore the dynamics of homestay or hosting and refugee well-being, offering valuable insights to inform the development of refugee homestay hosting models and practices across different host countries and geographical contexts. Employing the JBI framework as the methodological backbone of this scoping review represents a significant strength, ensuring rigor, transparency, and replicability in our approach [19,52]. The JBI framework is renowned for its comprehensive guidelines that facilitate the systematic identification, selection, and synthesis of a wide range of research evidence [19,52]. By adhering to this framework, we ensure that our review process is both methodical and inclusive, enabling us to encompass a broad spectrum of studies and thereby capture the multifaceted nature of the subject matter. Furthermore, the JBI framework’s emphasis on aligning the review objectives with the methodology used enhances the relevance and applicability of our findings. This approach not only strengthens the credibility of our review but also maximizes its potential to inform policy, practice, and future research, contributing valuable insights into the complex dynamics under investigation. One of the limitations is the potential exclusion of relevant studies due to language constraints and publication date. The decision to limit the review to English-language sources was made to ensure accessibility and relevance. While this constraint may exclude significant work in other languages, English is the dominant language in global academic discourse on refugee health, which minimizes potential bias. However, we acknowledge that non–English-language sources might offer additional perspectives, and this is a limitation that was added to the limitation section. As is the case in most scoping reviews, we did not aim to assess the quality of the selected sources. This underscores the need for a focused, in-depth examination of the selected studies in order to help expand on the preliminary insights provided in this scoping review. Additionally, the variability of studies across diverse cultural and geographical contexts might require context-specific research to grasp the intricacies of homestay or hosting and its impact on refugee well-being.

### Conclusions

This scoping review highlighted the complex relationship between homestay hosting and refugee well-being, uncovering the potential benefits, challenges, psychological and social impact, and factors that influence such arrangements. By examining the existing state of research, it illuminated the multifaceted nature of these hosting setups and their impact on both refugees and their host families. Our findings underscored the critical need for policies and practices that are deeply informed by an understanding of these intricate dynamics, aiming to effectively support the well-being of both refugees and their hosts. Grasping the intricate dynamics of homestay hosting arrangements is crucial for developing policies and programs that bolster the well-being of refugees and their host families. The review sheds light on the current knowledge landscape, identifying research gaps and potential ways to improve the homestay hosting experience for all parties involved.

## Appendix

Multimedia Appendix 1 PRISMA-ScR (Preferred Reporting Items for Systematic Reviews and Meta-Analyses extension for Scoping Reviews) fillable checklist.

Multimedia Appendix 2 Search strategy.

Multimedia Appendix 3 Data extraction table for included studies.

## Abbreviations

JBI: Joanna Briggs Institute
PRESS: Peer Review of Electronic Search Strategies
PRISMA: Preferred Reporting Items for Systematic Reviews and Meta-Analyses
PRISMA-ScR: Preferred Reporting Items for Systematic Reviews and Meta-Analyses extension for Scoping Reviews
