# Supplementary material for: Homestay Hosting Dynamics and Refugee Well-Being: Scoping Review
Source: Interact J Med Res. 2024 Nov 25;13:e58613. doi: 10.2196/58613 (PMC11629040; doi:10.2196/58613)
Supplement: Multimedia Appendix 2 [file ijmr_v13i1e58613_app2.docx]

Search conducted on CINAHL (EBSCO) on 13 December 2023.
AB ( Host* or (Host* N3 famil*) or homestay or cohabit* or foster* or (hospitality* N5 famil*) or accommo* )
AND
AB ( refugee* or asylum* or (displac* (person* or people*) )
AND ( impact or effect or influence or outcome or result or experience or perception or benefit or challenges )  
Language: English
Publication year: 2011-
Note: see table below for information on how the three-line search above was built up. Each search was first individually validated before being combined into a line of terms.

| **Search Development** | | | | | |
| --- | --- | --- | --- | --- | --- |
| Search Philosophy: find roots from within the research question What is known from the existing literature about the experiences [search group 3] of refugees [search group 2] with homestay hosting [search group 1]? | | | | | |
| Final search S8(abstract) AND S13(abstract) AND S23, limited to English and 2011 onwards | | | | | |
| Search | Group 1 host families and similar | Search | Group 2 refugees and similar | Search | Group 3 experiences and similar |
| S1 | Host* | S9 | Refugee* | S14 | Impact |
| S2 | Host* N3 famil* | S10 | Asylum* | S15 | Effect |
| S3 | Homestay | S11 | Displac* person* | S16 | Influence |
| S4 | Cohabit* | S12 | Displac* people* | S17 | Outcome |
| S5 | Foster* | S13 | S9 OR S10 OR S11 OR S12 | S18 | Result |
| S6 | Hospitality* N5 family* |  | *Living arrangement AND migrant** | S19 | Experience |
| S7 | Accommo* |  | Homestay AND integration | S20 | perception |
| S8 | S1 OR S2 OR S3 OR S4 OR S5 OR S6 OR S7 |  | Shared housing AND refugee | S21 | benefit |
|  |  |  |  | S22 | challenges |
|  |  |  |  | S23 | S14 OR … S22 |
