# Supplementary material for: Homestay Hosting Dynamics and Refugee Well-Being: Scoping Review
Source: Interact J Med Res. 2024 Nov 25;13:e58613. doi: 10.2196/58613 (PMC11629040; doi:10.2196/58613)
Supplement: Multimedia Appendix 3 [file ijmr_v13i1e58613_app3.docx]

**Appendix II: Data Extraction Table for Included Studies**

| **Study** | **Country/Setting/**  **Context** | **Participants**  **(nationality of refugees or Asylum seeker)** | **Aim/purpose** | **Methodology** | **Description of main results** |
| --- | --- | --- | --- | --- | --- |
| 1- Agblorti SKM. 2011. [24] | Ghana | Refugees from Liberia | Exploring the implications of humanitarian assistance to refugees for refugee-host relations. | Qualitative (in-depth interviews with two traditional authorities, a health professional, an opinion leader, assembly member, camp administrator) | The host community sacrifices significantly to accommodate refugees, facing issues with the UNHCR and its partners not compensating for the land and crops destroyed for camp construction. Despite the need to help refugee 'brothers and sisters,' the hosting community feels neglected, with resources diverted to refugees, negatively impacting locals. There's a call for the host community to benefit from interventions aimed at refugees. The UNHCR's neglect and the unfair distribution of resources have strained relations between hosts and refugees, with the former unable to benefit from the aid provided to the latter, potentially harming repatriation efforts and fostering strained relationships. |
| 2-Al-Saqaff N. 2024.[31] | Netherlands | 161 Displaced Ukrainians who fled to the Netherlands after the Russian invasion. | To investigate the host national contact of displaced Ukrainian in the Netherlands. | Quantitative (correlational) | The quality and quantity of contact with host nationals did not uniformly predict mental health among refugees; however, more contact correlated with better mental health outcomes. Female refugees benefited from increased contact with co-nationals, showing less distress. Not all displaced Ukrainians aimed to stay in the Netherlands. A notable finding was the positive link between perceived discrimination and mental health among those who responded in Ukrainian, suggesting that discrimination might reinforce ethnic identity and, paradoxically, improve mental health during uncertain times like war and displacement. |
| 3-Alrawadieh et al 2023.[33] | Slovakia | 160 valid surveys of Ukrainian refugees in Slovakia and 25 in-depth interviews with Slovakian individuals hosting refugees | To examine the role of hospitableness towards refugees, as embraced by local hosts, in engendering positive social outcomes, including fostering favourable attitudes and empathy towards refugees, satisfaction from hosting refugees in private dwellings and advocacy for hosting them. | Mixed method (sequential quantitative-qualitative design through surveys and in-depth interview) | Informants largely reported positive hosting experiences, although satisfaction waned over time due to various constraints. Direct contact with refugees allowed hosts to gain insight into their often-traumatic backgrounds, fostering genuine empathy. Factors like common interests, religious ties, cultural proximity, and gender significantly influenced hosting behavior. The study underscores a positive correlation between hosting satisfaction and advocacy for refugee hosting. |
| 4-Altinay et al 2023.[34] | Slovakia | 135 Ukrainian refugees hosted by locals in Slovakia | To test a conceptual model linking hospitableness, social inclusion, self-esteem, loneliness, psychological distress, psychological resilience, and subjective well-being. | Quantitative (non-experimental) | The findings suggest that hospitableness plays a key role in refugee social inclusion, creating a welcoming atmosphere that builds emotional ties and cultural connections with hosts, thus enhancing societal cohesion. Employment boosts refugees' self-esteem and overall well-being. Psychological resilience is essential for fully benefiting from hospitableness, with more resilient refugees achieving better social integration. However, hospitableness has a limited impact on internal issues like psychological distress and does not significantly affect the deeply traumatized, especially women separated from their families, highlighting the nuanced effects of external support on personal challenges. |
| 5-van Dijk et al 2021.[35] | Netherlands | 53 Syrian refugees and 51 hosts | To investigate and understand the effectiveness of Social Innovation (SI) called TakeCareBnB which facilitates refugee integration by letting refugees temporarily reside with local residents. | Qualitative (grounded theory) | TakeCareBnB significantly enhances refugee integration across all areas outlined by Agar and Strang's framework. It encourages volunteer work as a pathway to employment, aids in securing permanent housing, and improves health outcomes through emotional support and privacy. Refugees gain more access to education, establish social connections with locals, and integrate into host family life, which fosters cultural and language learning. While staying with locals reduces bonds with like-ethnic groups, it strengthens family connections, promoting integration. However, challenges like cultural clashes and privacy concerns need professional support for resolution. This model underscores integration as a mutual process, highlighting the importance of social bridges and balanced social bonds. |
| 6-Sirriyeh A. 2013.[19] | United Kingdom | 133 foster carers, case  studies with 23 foster carers and 21 young people in  their care (Afghanistan ) | To describe the fostering experiences of young people and foster carers.  To identify specific features of the fostering task in the broad resettlement needs of young people.  To assess the support provided to young people and preparation, training, and support of foster carers.  To identify factors that facilitated or constrained the making of placements. | Qualitative (case studies analysis and in depth interviews) | In fostering contexts, relationships vary: 'Like-family' fosters 'family-like' bonds through welcoming actions; 'Guests' maintain respectful, yet less close relationships due to differing interests; 'Lodgers' experience tension and lack of belonging, limiting hospitality. Positive outcomes arise when young refugees participate in household culture and develop trusting, reciprocal relationships. Challenges include overcoming prejudices and the professionalization of care, which can hinder the display of familial affection. Integration and a sense of belonging are crucial, yet complex, influenced by the dynamics of each unique fostering situation. |
| 7-Ran GJ, Join-Lambert H. 2020.[11] | France | 7 from Syria,  6 from Tibet,  4 from Bangladesh,  3 from Zimbabwe  (20 refugees, 10 hosting family  members and 4 social workers) | To examine the actual influences of family hosting on refugee integration by using the case of France, who faces vital challenges of refugee reception and settlement. | Qualitative (In depth interviews) | Enhanced language skills, cultural understanding, physical and mental health, employment, and educational opportunities result from host engagement. However, challenges include reduced independence and privacy concerns, highlighting the need for balanced relationships to avoid potential conflicts. |
| 8-Monforte et al 2021.[37] | Europe (Britain, France and Italy.) | 47 volunteers who host refugees in Britain, France and Italy. | To explore the motivations and experiences of individuals who practice private hospitality by hosting refugees in their homes. | Qualitative (In depth interviews) | Hosts motivated by social change and critique of government refugee policies feel a duty to help, treating refugees as family and seeking emotional connections. Unmet expectations for reciprocated affection can lead to emotional strain and potential exclusion for refugees. Hosting offers insights into immigration injustices, cultural exchange, and family bonding. |
| 9-Merikoski P. 2021[30] | Finland. | 30 hosts, Syrian, Iraq, Afghanistan | To examine the home accommodation of asylum seekers in Finland from the perspective of hosts’ affective experiences of living through the asylum process from proximity. | Qualitative (In depth interviews) | Asylum seekers' experiences deeply intertwine with their hosts' daily lives, affecting them both sensorially and emotionally. Technology's role in connecting refugees with their families during conflict blurs the lines between hosts' homes and the war outside, making hosts more than mere observers and emotionally involving them in the refugees' suffering. The trauma is further compounded when refugees face deportation due to government policies, impacting the hosts' sense of security.  Despite these challenges, hosting refugees is rewarding for many, offering opportunities to broaden perspectives, meet new people and cultures, and help those in need, making it an irreplaceable experience. However, some hosts feel a lack of emotional connection, leading to feelings of restriction and disappointment. |
| 10-Hebbani et al 2016.[27] | Australia | 455 comments from six online Australian news  sources | To explore the media framing of, and online readers’ comments on, the announcement of the Government’s Community Placement Network (CPN) homestay initiative. | Qualitative Leximancer text  analysis method | Media coverage on asylum seekers often focuses on detention and payments to host families, using sensational language and incorrect terminology, which frames asylum seekers negatively and influences public opinion. The use of terms like "illegal immigrants" in comments with negative sentiment and incorrect references to asylum seekers as "refugees" contributes to their stigmatization. The media's selective framing and demonization of the CPN initiative further exacerbate misunderstandings and negative perceptions, despite efforts by some to highlight the initiative's benefits. This incorrect and biased representation promotes discrimination, underscoring the media's power in shaping public views. |
| 11-Aparna K, Schapendonk J. 2020.[38] | Not specified; focusing more on the dynamics and the challenges within a local migrant-support organization. | Not specified; focusing more on the dynamics and the challenges within a local migrant-support organization. | The aim of this study is to critically examine and challenge existing empirical studies on refugee support, hospitality, and migration. | Opinion paper | The study challenges the rigid definitions of "hosts" and "guests" in the context of asylum, advocating for a dynamic understanding that encompasses border-crossings, emotional labor, and the fluid political geographies of asylum. It introduces "asylumscapes" to describe the evolving nature of hospitality practices and calls for a recognition of the complex interactions in asylum situations. The research emphasizes the need for a process-oriented geographical approach to better understand migration's impact on social and political structures. It also highlights the role of universities in challenging traditional views on citizenship and mobility and encourages them to engage in the exchange of knowledge related to asylum dynamics. Overall, the study promotes a more nuanced and inclusive approach to hospitality and asylum, emphasizing engagement and understanding of the complexities involved. |
| 12-Baban et al 2017.[20] | Germany | Refugees who come to Berlin to seek asylum (nationality not specified) | To address the question of reconciling cultural plurality with communitarian concerns regarding maintaining long-established traditions and norms of those societies, by rethinking citizenship with the help of radical cosmopolitanism | Qualitative (case study) | Radical cosmopolitanism redefines the relationship between self and others, moving away from traditional hierarchies that view hosts as superior and refugees as inferior. It emphasizes everyday practices and lived experiences with newcomers, prioritizing social interaction over mere charity, thus challenging traditional citizen/non-citizen divides and advocating for equality and the obligation to protect refugees. This approach fosters equal exchange and togetherness, breaking down exclusionary barriers. Examples of radical cosmopolitanism in action include community initiatives like movie nights in refugee camps and kitchen projects, showcasing how embracing cultural, religious, and other diversities through everyday interactions can lead to a more inclusive society. |
| 13-Brinker LD. 2021.[6] | Spain | 30 in‐depth interviews with hosts, refugees, intermediaries, and social workers in Catalonia (Spain) | exploring the role of intermediaries in host-refugee dyads; examining whether they can contribute to the formation of local social capital and compensate for potential power differentials in refugee-host relationships. | Qualitative (In depth interviews) (ethnography) | Refugees viewed intermediaries as crucial bridges within the hosting network, offering flexibility, personalized support, and acting as a safety net by easing initial co-living adjustments, offering advice, and emotional support. However, intermediaries sometimes reinforced power imbalances by overstepping boundaries, and their close relationships with hosts could compromise their neutrality. Additionally, intermediaries often withdrew their involvement once they perceived themselves as no longer needed. |
| 14-Caron C. 2017.[1] | NA | Sri Lanka , Gambia, Haiti, the Democratic Republic of Congo, Lebanon, Ethiopia | To describe factors that shape the hosting experience. | Opinion paper | Uncertainty about the length of stay and the potential of overstaying creates anxiety for hosting families. The presence of children can destabilize the hosting dynamic due to conflicts among kids or feelings of resentment by refugee children towards their own parents, influenced by comparisons with the host family's children. The necessity to share space and resources leads to challenges; a significant portion of hosted families eventually move out to separate accommodation due to unsustainable relationships or insufficient space. Economic constraints can also make sharing resources a source of conflict, though participation in household chores by hosted family members can mitigate feelings of indebtedness and foster a sense of contribution. |
| 15-Caron CM. 2019.[2] | NA | literature review | To identify and summarize ten aspects that shape the hosting environment and its associated support programs. | literature review and gray literature | Ten factors influencing hosting relationships include the uncertainty of stay length, the physical size and location of the hosting space, accommodation costs, family size potentially causing overcrowding, the presence and behavior of children, the socio-economic status of the host family, sharing of living space and resources, and communal activities. Humanitarian actors are encouraged to develop context-sensitive support plans addressing these factors, such as specifying sleeping arrangements, utility access, and kitchen use. Recommendations include providing aid to both host and guest families to prevent conflicts, offering counseling to discuss challenges and solutions, and implementing interventions like paying for children's schooling and providing employment training to lessen financial strain and foster social integration. These measures aim to support displaced families in exercising their rights and making informed choices about their lives. |
| 16-Hebbani et al 2016.[40] | Australia | 24 men and women from all over Australia were interviewed (host families) | To investigate host families’ motivations for volunteering into the home accommodation scheme and their resulting experiences. | Qualitative (In depth interviews) | People became hosts out of empathy, sympathy, and protest against detaining asylum seekers. Hosts benefited by learning about new cultures, while the community saw reduced prejudices and gained new skills from refugees. Refugees benefited from increased self-esteem, language skills, and supportive networks. Challenges included organizational issues, cultural and language barriers, and psychological distress among guests. Guests struggled with adapting to new climates and felt isolated without access to ethnic communities. Hosts faced skepticism from family and friends, who sometimes perceived them as risky or traitorous for supporting refugees. |
| 17-Trenson et al 2023.[29]merk | Belgium | 27 Unaccompanied Refugee Minors (URM)  cared for in family foster care from Afghanistan, Syria, Congo, Nigeria, Eritrea, China, Iraq | To examine how unaccompanied refugee minors (URM) develop in Flemish foster care with regard to behavioural problems and trauma symptoms.  To investigate if the development of URM is associated with the cultural background of the foster families. | Quantitative | About 60% of Unaccompanied Refugee Minors (URM) show high trauma levels, needing social and psychological support. Lack of support risks placement breakdown and foster care development. Despite the highly supportive environment, URMs require specialized psychological help due to low resilience. While foster care offers stability and family connectedness, enhancing resilience, kinship care often fails to do so because of additional stressors. Social support and cultural connections in foster care reduce trauma symptoms, serving as protective factors and promoting resilience. Interaction with local peers and adults reduces behavioral issues, aiding integration and future outlook. |
| 18-Lauterbach K. 2021.[36] | Uganda | 88 interviews Congolese refugees and 13 church members and pastors | To analyze hybrid forms of hosting in a faith-based context and to discuss the implications of this for how guest and host categories are perceived. | Qualitative (In depth interviews) | Refugee pastors and church members play distinct roles in hosting refugees. Pastors offer their homes, counsel, and act as liaisons with formal systems, driven by a sense of Christian duty. Church members provide practical aid like accommodation and essentials, though interactions with refugees are less familial, marked by a sense of strangerhood and distrust. Pastors, viewed with high social regard, may exchange spiritual services for support, reflecting gratitude and devotion to God. Their actions are seen as extensions of faith, demonstrating compassion and sacrifice, with pastors positioned as key mediators of divine power within social hierarchies. |
| 19-Messiah et al. 2016.[21] | USA | 416 surveys with Haitian participants | To examine the possible mental health effects on the host families of 2010 Haiti earthquake refugees. | Quantitative | Hosting refugees from the 2010 Haiti earthquake led to increased depression symptoms and mental health disorders in hosts, possibly due to reactivating their traumatic memories through the refugees' disaster stories. While hosting offers a chance for kindness and altruism, it also poses risks to the hosts' mental health, exacerbated by the strain of shared living spaces and resources. |
| 20-Bassoli M, Luccioni C. 2023.[42] | NA | Literature Review. | To reflect on the political issues that homestay accommodation for refugee raises. | systematic literature review) | Homestay accommodation offers advantages like central location, comfort, and no schedule restrictions, fostering shared experiences, bonding, and a support network. It enhances language, intercultural skills, education, employment opportunities, and emotional support. However, hospitality inherently creates an unequal relationship, positioning refugees as inferior and reducing their agency, often leading to a debt of gratitude. Homestays can disrupt host family intimacy, strain relationships, cause psychological fatigue, and disappoint if expectations aren't met. Refugees may find the arrangement demanding and intrusive. |
| 21-Mahieu R, van Caudenberg R. 2020.[9] | Belgium | between one and three inter-  views were conducted with 23 refugees and 17 buddies Young accompanied refugees (aged 17-23) in Belgium (nationality unspecified) | To discuss an urban programme that offers young accompanied refugees cohabitation with young locals (aged 20-30) during a period of 1-2 years in Antwerp (Belgium) in small-scale collective housing units. | Qualitative (In depth interviews) | Communal living offers social support to refugees through tangible support like gifts and shared items, informational and emotional support via companionship, and mental well-being. It creates a dependency and gratitude towards the hosts, extending the host's social network to refugees and providing a low barrier for seeking help. This informal, befriending nature facilitates mutual learning beyond the duration of the stay, enhancing language and cultural understanding through everyday activities and discussions, contrasting with formal education's hierarchical structure. Communal living fosters two-way cultural exchange and challenges existing attitudes and behaviors, promoting self-awareness and respect, especially in gender-mixed settings, leading to discussions on gender roles and cultural perceptions. |
| 22-Merikoski P, Nordberg C. 2023.[32] | Finland | 30 interviews with local hosts who accommodated asylum seekers in Finland (Iraq, Syria, Afghanistan, Ukraine) | To investigate how the racialized and gendered public discourses on asylum-seekers are challenged and reproduced in home accommodation. | Qualitative (In depth interviews) | Asylum seekers in Finland often find housing through informal channels like pro-asylum activism or volunteering, with the Home Accommodation Network playing a facilitating role. Hosts typically join through activism, establishing natural connections with potential guests. Despite racialized public discourse, hosts' preferences vary, challenging stereotypes. While requests for specific guest profiles like "woman with a small child,” may reveal biases, hosts often claim to have no preferences, indicating the complexity of host-guest dynamics. Selection criteria prioritize personal compatibility and shared values over perceived deservingness, with class, gender, and race influencing relationships and daily interactions. Cultural and educational differences, spatial organization, gender boundaries, and the portrayal of Finnish life and gender equality by hosts all contribute to shaping experiences and promoting integration. The study highlights the nuanced nature of home accommodation, emphasizing the role of personal relationships and shared values in shaping the experiences of both hosts and asylum seekers. |
| 23-Bassoli M, Campomori F. 2022.[3] | Italy | 8 case studies with refugees and asylum seekers in Italy. 23 semi-structured interviews with project managers, civil servants, scholars, or policymakers | To develop a theoretical framework to identify policy beneficiaries and policy targets by looking at policy goals | Qualitative (In depth interviews) | Homestay projects often prioritize migrant well-being and social inclusion. In Asti, migrant hosts provide services on behalf of the public authority, emphasizing a homocultural approach. Milan showcases the city as a safe haven, with hosts and guests serving as volunteers for social inclusion. In Fidanza and Parma, intercultural dialogue is key, with hosts supporting guest autonomy. These projects aim for societal change alongside migrant integration, benefiting both hosts and guests. |
| 24- Merikoski P. 2022.[31] | Finland | 30 interviews with hosts who accommodated asylum seekers Individuals seeking asylum in Finland (Iraq, Syria, Afghanistan, Ukraine) | To discuss hospitality towards asylum seekers in local homes is one of the pro-asylum mobilizations across Europe. | Qualitative (In depth interviews) | Individuals become hosts motivated by moral shock from media coverage, leading to a desire to open their homes. Hosting asylum seekers fosters mistrust in the Finnish government and transforms perceptions of the country from safe to hostile. Strong bonds form between hosts and asylum seekers, fostering spatial and emotional closeness that blurs need over time. Home accommodation facilitates connections with locals and urban spaces, contrasting with remote asylum centers, enabling migrants' self-organization and integration. Despite its benefits, immigration authorities often view home accommodation negatively, leading to further distrust. Hosts see their homes as sites of political agency, where they resist negative societal developments through hospitality, symbolizing power in the context of migration restrictions. |
| 25- [Khawaja, N](https://eprints.qut.edu.au/view/person/Khawaja,_Nigar.html) & [Wotherspoon, J](https://eprints.qut.edu.au/view/person/Wotherspoon,_Jane.html), (2015).[28] | Australia | (N = 142) hosts completed online questionnaire for demographic variable and attitudes towards cultural diversity and acculturation. Followed by two focus groups. | This study compared those who volunteered to assist asylum seekers with general members of the community to explore the impact attitudes towards cultural diversity and demographic factors had upon willingness to support asylum seekers | Mixed methods | Volunteers’ attitudes did not impact on willingness to support asylum seekers Analyses on the combined groups indicated age, education not having a strong religious affiliation or enhanced Australians’ positive attitudes to cultural diversity. |
